# Receptor Positive Breast Lesions and Status of Axillary Lymph Node

**DOI:** 10.7759/cureus.50645

**Published:** 2023-12-16

**Authors:** Vinod Kumar Singhal

**Affiliations:** 1 General Surgery, PRIME Hospital, Dubai, ARE

**Keywords:** human epidermal growth factor receptor-2, hormone receptor-positive breast cancer, breast cancer, metastasis, axillary lymph node

## Abstract

Objective

This study aimed to examine the axillary pathological condition through the development of an assessment framework for hormone receptor-positive breast cancer subsequent to neoadjuvant chemotherapy (NAC). Furthermore, the primary objective of this study was to examine the association between axillary status and breast tumors that are positive for hormone receptors following neoadjuvant chemotherapy.

Methodology

The present retrospective investigation encompassed a cohort including 300 individuals who were administered neoadjuvant chemotherapy before receiving surgical intervention. The data collection period for this study was from September 2021 to December 2022. All patients received neoadjuvant chemotherapy, per the guidelines established by the National Comprehensive Cancer Network (NCCN). We divided patients into two distinct groups: a test set of 250 patients and a validity set of 50 patients. Patients with no evidence of lymphoid involvement underwent a biopsy of sentinel lymph nodes (SLNB) and would have undergone axillary dissection if the biopsy results had indicated positive findings. A logistic regression analysis was employed to investigate the variables linked to the presence of residual positive axillary lymph nodes in the test set. Subsequently, a multivariate analysis was conducted on the variables that exhibited a p-value below 0.2 in the univariate study. In addition, a value of 1 was assigned to all risk factors to construct a comprehensive correlation prediction model.

Results

The participants included in this study had a mean age and body mass index (BMI) of 46.24 ± 9.1 years and 25.8 ± 2.5 kg, respectively. The present investigation examined the presence of pathological axillary metastases in a cohort of 188 patients, which accounted for 62.55% of the total sample, by utilizing core-needle biopsy. Furthermore, the incidence rates of individuals presenting with clinical T1 were reported as 14.6%, while 55.2% cases of T2, 17.8% cases of T3, and 13% cases of T4 tumors were reported, respectively. Of the overall occurrences, the prevailing histological subtype was invasive ductal carcinoma, accounting for 91.4% (222 out of 243) of the cases. Multiple criteria were identified as independent predictors of the presence of residual positive axillary lymph nodes. The factors under consideration encompass lymphovascular invasion (odds ratio= 7.108; 95% breast cancer stage (odd ratio = 5.025; 95%, HER2 negativity (odd ratio= 2.997), low Ki-67 expression (odd ratio = 4.231), and suspected positive axillary lymph nodes before surgical intervention.

Conclusion

The present study presents a novel prediction model that integrates imaging and pathology data, aiming to assist patients and healthcare practitioners in evaluating the efficacy of NAC for hormone-receptor-positive breast tumors. The model holds particular significance for individuals who exhibit clinical positivity in their lymph nodes (LNs). Consequently, the model has the potential to provide guidance for the management of axillary lymph nodes and prevent unnecessary dissection.

## Introduction

Breast tumors are more frequently observed in women than lung cancer, making them the most common type of cancer among the female population [1]. Breast cancer is commonly categorised into three distinct subtypes: triple-negative breast cancer (TNBC), human epidermal growth factor receptor II (HER2)-positive breast cancer, and hormone receptor-positive (HR+) breast cancer. The subgroups of hormone receptor-positive (HR+) breast tumours are Luminal A, Luminal B HER2-negative, and Luminal B HER2-positive. These subgroups shown significant sensitivity to endocrine therapy following surgical procedures [2,3]. Nevertheless, there is a dearth of empirical data about the utilisation of neoadjuvant chemotherapy for different types of breast tumours.

Neoadjuvant chemotherapy (NAC) is a well-established treatment modality that is commonly used for the management of incurable malignancies. Its primary objectives include reducing the size of cancerous tumors, increasing the likelihood of breast-conserving surgery (BCS), and minimizing the incidence of axillary metastasis [3-4]. The efficacy of neoadjuvant therapy has been demonstrated, even when the initial recommendation for axillary dissection was later substituted with lymph node sentinel biopsy [4]. The evaluation of pCR signifies pathological complete response is of considerable significance within this specific framework, serving as a crucial measure for assessing the efficacy of NAC. Patients who achieved a pathological complete response (pCR) in both the breast and axillary lymph nodes (LNs) after receiving neoadjuvant chemotherapy (NAC) demonstrate improved survival outcomes and longer periods of disease-free survival, regardless of their initial clinical condition [5,6]. However, it is essential to note that the rate of pathological complete response (pCR) varies based on the subtype of breast carcinoma. Breast cancer, characterized by hormone receptors, specifically, exhibits a decreased incidence of pathological complete response (pCR) in comparison to breast cancer with human epidermal growth factor receptor 2 (HER2+) and triple-negative breast cancer (TNBC). This is even though breast tumors with hormone receptor positivity (HR+) generally have a more favorable overall prognosis [7,8]. Consequently, a decrease in the efficacy of NAC has been shown in hormone receptor-positive (HR+) breast cancer, leading to ongoing debates regarding the appropriateness of utilizing NAC in this particular patient population.

The primary objective of NAC is to attain pathological complete response (pCR), which is a widely pursued outcome. Patients, even those initially considered for total mastectomy, should still seek breast-conserving surgery (BCS) following successful NAC [9]. However, there is a limited number of studies that have assessed the axillary pathological complete response (pCR) rate, especially for people suffering from hormone deficiencies receptor-positive (HR+) breast cancer who underwent NAC [9-11]. There are currently no reliable approaches for accurately predicting the condition of an axillary lymphatic node (ALN) before surgical intervention. Therefore, this study was designed to investigate the role of neoadjuvant chemotherapy for patients with hormone receptor breast lesions and develop a framework for accessing the axillary pathological conditions to minimize the ratio of breast surgery. Furthermore, this study also examined the association between axillary status and breast tumors that are positive for hormone receptors following neoadjuvant chemotherapy.

## Materials and methods

The present retrospective investigation encompassed a cohort including 300 individuals who were administered neoadjuvant chemotherapy before receiving surgical intervention. The data collection period for this study was from September 2021 to December 2022. The study was conducted after attaining ethical approval from the general surgery department of Prime Hospital, Dubai (001/2020/CREC/PH). Before and during the administration of neoadjuvant therapy, axillary ultrasounds were performed on all patients. For this research, the term "clinically node-positive breast cancer" pertains to instances when the presence of axillary lymph node metastases is either pathologically verified or clinically suspected, as per the established definition. The phrase "axillary pathological complete response" was subsequently used to indicate the lack of metastases in the pathology of surgical procedures. The present investigation employed a random sample technique to assign the recruited individuals to two distinct groups: the experimental group (n = 250) and the validation group (n = 50).

We formed exclusion criteria for the study after a comprehensive evaluation of obtained clinical results and patient imaging data. Inclusion criteria were applied to the study population, resulting in the exclusion of specific patient groups. The analysis excluded certain groups of individuals, including those who were clinically asymptomatic (n=60), those who did not have access to imaging data, specifically axillary ultrasound, either before or following neoadjuvant treatment (n=50), those who had distant metastases at the beginning of the study (n=2), those with a previous history of breast cancer (n=13), and those with undetermined malignancy neoadjuvant chemotherapy data (n=10). According to the criteria set forth by the National Comprehensive Cancer Network (NCCN), all patients were administered neoadjuvant chemotherapy consisting of anthracycline and taxol regimens [12]. Among the 300 patients included in the study, 57 (18.93%) patients achieved a complete axillary pathological response following the administration of neoadjuvant chemotherapy. Patients with no evidence of lymphoid involvement underwent a biopsy of sentinel lymph nodes (SLNB) and would have undergone axillary dissection if the biopsy results had indicated positive findings [13].

The study collected clinical and pathological data, including variables age, BMI, menopause position, initial medical and final malignancy phase, preliminary medical and ultimate nodal stage, histopathological type, surgery for breast cancer method, axillary surgical technique, pathologic cancer phase, and node phase are all factors to consider. Despite this, the current investigation also collected data on the expression of Ki-67, the HER2 status, neoadjuvant chemotherapy regimen, the therapy cycle, and lymphovascular diseases of all eligible patients. We investigated the rate of axillary pathologic complete response (pCR) among patients who had received neoadjuvant chemotherapy as an independent factor.

All the collected data was analyzed using version 25 of the IBM SPSS analytics software. A logistic regression analysis was employed to investigate the variables linked to the presence of residual positive axillary lymph nodes within the test set. Subsequently, a multivariate analysis was conducted on the variables that exhibited a p-value below 0.2 in the univariate analysis. In addition, a value of 1 was assigned to all risk factors to construct a comprehensive correlation prediction model. Hence, the cumulative risk score for every patient in the test set was calculated by aggregating the applicable risk ratings [14]. The Hosmer and Lemeshow goodness-of-fit test was employed to assess the calibration and discrimination properties. A p-value below 0.05 signifies the statistical significance of the tests.

## Results

The study included a total of 300 people who were diagnosed with hormone receptor-positive (HR+) breast cancer. Table 1 presents these patients' clinical and pathological characteristics before and after NAC. The participants included in this study had a mean age and BMI of 46.24 ± 9.1 years and 25.8 ± 2.5 kg, respectively. The present investigation examined the presence of pathological axillary metastases in a cohort of 188 patients, which accounted for 62.55% of the total sample, by utilizing core-needle biopsy. Furthermore, the incidence rates of individuals presenting with clinical T1 were reported as 14.6%, while 55.2% cases of T2, 17.8% cases of T3, and 13% cases of T4 tumors were reported, respectively. Among these cases, invasive ductal carcinoma was the prevailing histological subtype, accounting for 91.3% (274 out of 300) of the total occurrences. Results revealed a pathological complete response (pCR) in 7.8% (24 out of 300) patients having a main tumor. Conversely, for axillary nodes, the proportion of patients exhibiting pCR was 19.9% (60 out of 300). Therefore, it is apparent that the achieved rate of pathological complete response (pCR) by the use of NAC in hormone receptor-positive (HR+) breast cancer is less than ideal for both the original tumor and the axillary lymph nodes (Table 1).

Additionally, it is worth noting that there was no significant disparity in the distribution of the variables observed between the test and validation sets. This lack of statistical difference was confirmed by doing a hypothesis test, with all p-values exceeding 0.05. Results also depicted that 68 (22.6%) patients in the test set attained axillary pathological complete response (pCR). In contrast, the remaining 72.8% (182 out of 250) had residual positive axillary lymph nodes (LNs) (Table 1).

**Table 1:** Clinical characteristics of patients before treatment modality

Parameters	Test set (N=250)	Validity set (N=50)	Total (N=300)	p-value
Age in years	50.98 ± 11.23	51.34 ± 8.7	46.24 ± 9.2	0.67
Body mass index (in kg)	23.65 ± 4.78	24.78 ± 4.4	25.8 ± 2.5	0.52
Menopausal status				0.289
Post menopausal	133 (53.2%)	23 (46%)	156	
Premenopausal	117 (46.8%)	27 (54%)	144	
Clinical stage of lymph nodes (N)				0.413
Stage 3	38 (14.9%)	7 (14%)	47	
Stage 2	78 (31.2%)	20 (40%)	98	
Stage 1	134 (54.3%)	23 (46%)	157	
Clinical stage of tumor (T)				0.883
Stage 4	31 (12.4%)	9 (18%)	40	
Stage 3	31 (12.4%)	7 (14%)	38	
Stage 2	150 (60%)	29 (58%)	179	
Stage 1	38 (14.9%)	5 (10%)	43	
ypN stage				0.551
Stage 3	37 (14.8%)	13 (26%)	50	
Stage 2	65 (26%)	11 (22%)	76	
Stage 1	97 (38.8%)	19 (38%)	116	
Stage 0	51 (20.4%)	7 (14%)	58	
ypT stage				0.491
Stage 4	7 (2.8%)	7 (14%)	14	
Stage 3	14 (5.6%)	3 (6%)	17	
Stage 2	84 (33.6%)	16 (32%)	100	
Stage 1	128 (51.2%)	22 (44%)	150	
Stage 0	17 (6.8%)	2 (4%)	19	
Histological type				0.32
Invasive ductal carcinoma	228 (91.2%)	46 (92%)	274	
Invasive lobular carcinoma	7 (2.8%)	3 (6%)	10	
Others	15 (6.7%)	1 (2%)	16	
Lymph node surgery				0.145
Axilary lymph node dissection	246 (98.4%)	49 (98%)	295	
Sentinel lymph node biopsy	4 (1.6%)	1 (2%)	5	
Breast surgery				0.221
Breast conserving surgery	85 (34%)	16 (32%)	101	
Mastectomy	15 (6%)	7 (14%)	22	

The initial analysis focused on a single variable, revealing that the clinical N2 stage was observed more frequently in cases where residual positive axillary lymph nodes were present, as opposed to instances where an axillary pathological complete response was achieved (p<0.2). Furthermore, results indicated that individuals who had undergone neoadjuvant chemotherapy and still had positive axillary lymph nodes reported a higher likelihood of exhibiting suspected nodules during ultrasound examination before surgery (p<0.2). In conclusion, the results obtained from the pathological examinations revealed a notable correlation between decreased levels of Ki-67, HER2 negative, histological grade III, and lymphovascular invasion and residual positive axillary lymph nodes (p< 0.2). Overall, there were no significant differences identified between the two groups in terms of age, clinical T stage, histologic type, and grade, axillary surgery, as well as NAC regimens and cycles (p> 0.2) (Table 2).

**Table 2:** Univariate regression analysis for test set group (N=250)

Parameters	Odd ratio	At 95% confidence interval		p-value
Age	0.978	0.942 to 1.031		0.784
Clinical stage of node (N)					
Stage 3	3.987	1.098 to 21.821		0.029
Stage 2	2.421	1.034 to 7.350		0.032
Stage 1	Reference	-	-	
Clinical stage of tumor (T)					
Stage 4	0.717	0.141 to 3.981		0.321
Stage 3	1.798	0.299 to 12.88		0.639
Stage 2	0.498	0.186 to 1.971		0.856
Stage 1	Reference	-	-	
Histological type					
Others	0.499	0.138 to 2.786		0.652
Invasive ductal carcinoma	Reference	-	-	
Neoadjuvant chemotheraphy regimes					
Taxol	0.399	0.076 to 2.576		0.476
Anthracycline + Taxol	1.123	0.513 to 2.998		0.980
Anthracycline	Reference	-	-	
Ultrasound of lymph nodes					
Positive	5.032	1.635 to 9.841		0.0001
Negative	Reference	-		-
Lymphovascular invasion					
Positive	4.023	1.298 to 10.032		
Negative	Reference	-	-	
Breast surgery					
Breast conserving surgery	5.371	1.795 to 14.01		0.0002
Mastectomy	Reference	-	-	
Neoadjuvant chemotheraphy cycle					
Less than 6	0.681	0.076 to 7.412		0.6822
4 to 6	0.662	0.056 to 5.231		0.965
Less than 4	Reference	-	-	
Human epidermal growth factor receptor 2 (HER2)					
Positive					
Negative	1.982	1.023 to 6.089		0.032
Ki-67					
< 14	3.276	1.498 to 8.063		0.002
≥ 14	References	-	-	
Histological grade					
III	2.391	0.792 to 6.732		0.154
I/III	Reference	-	-	
ypT stage					
1 to 4	1.792	0.631 to 7.142		0.489
0	Reference	-	-	

According to the multivariate analysis findings (Table 3), various characteristics were identified as independent predictors of residual positive axillary lymph nodes. The factors considered in this study include lymphovascular invasion (odds ratio = 7.108, HER2 negativity (odd ratio = 2.997), low Ki-67 expression (odd ratio = 4.231), suspected positive axillary lymph nodes before surgery (odd ratio = 5.113), histological grade III (odd ratio = 5.109), and breast-conserving surgery breast cancer stage (odd ratio = 5.025) (all with p < 0.05) (Table 3).

**Table 3:** Multiple regression analysis for test set group (N=250)

Variables	Odd ratio	At 95% confidence interval	p-value
Ultrasonography of positive lymph node	5.113	1.62 to 14.238	0.0006
Lymphovascular invasion (positive)	7.108	1.682 to 23.156	0.0017
Histological grade (III)	5.109	1.422 to 8.562	0.00015
Ki-67 (<14%)	4.231	1.394 to 9.982	0.0014
Human epidermal growth factor receptor 2 (negative)	2.997	1.098 to 9.231	0.009
Breast conserving surgery)	5.025	1.289 to 18.012	0.002

Subsequently, researchers developed a predictive model using the abovementioned factors. All risk factors were assigned a value of 1 to create a complete correlation prediction model, resulting in a total risk score between 0 and 6. The Receiver Operating Characteristic (ROC) analysis was next employed to demonstrate the favorable discriminative performance of the prediction model, as evidenced by an Area Under the Curve (AUC) value of 0.837. Furthermore, researchers used the Hosmer-Lemeshow test to check the calibration of the prediction model. The results yield a validity statistic of 0.556 (P < 0.001), indicating the model's good recognition and correction abilities.

Furthermore, the area under the receiver operating characteristic curve (AUC) was 0.813 after completing internal validation. This AUC value suggests that the model exhibits high calibration ability. The Hosmer-Lemeshow test showed a value of 0.937 and a statistical significance value of less than 0.001, showing good calibration abilities. In the context of the test and validation sets, a positive correlation exists between higher total risk scores and an increased likelihood of residual positive axillary lymph nodes.

## Discussion

In our study, we found that the overall rate of attaining a pCR indicates pathological complete response in the breast region among HR+ cancer sufferers who had NAC was 6.6%. Furthermore, we discovered that 18.9% of patients in the axillary area achieved a pathological complete response. Thus, the results suggested that the pathological complete response (pCR) rate in hormone receptor-positive (HR+) patients after NAC would not be as high as it could be, which is in line with prior research [15,16]. The present study examined the correlation between abnormal axillary status and pre-surgical parameters to develop a predictive model for the probability of axillary pathological remission in HR+ breast tumors. The prediction model demonstrated satisfactory performance in assessing the axillary response following NAC. Multivariate and univariate analysis assists in the identification of six risk variables that contribute to a negative outcome in the axilla following NAC. Ultrasonography (US) remains the prevailing method for acquiring imaging evidence of axillary lymph nodes to assess axillary response after NAC. Furthermore, it is essential to note that extensive research has established a substantial relationship between lymphovascular invasion (LVI) and the presence of distant metastases in several types of solid tumors. Moreover, it serves as an adverse prognostic indicator for both survival rates and the likelihood of recurrence [17]. 

Prior research endeavors have predominantly concentrated on recording the phenomenon through surgical pathology reports, with a restricted comprehension of its molecular biology. Previous research has examined the potential suitability of persons with positive LVI for adjuvant treatment to ascertain the prognostic implications for survival in cases of early gastric cancer [18,19]. Similarly, results indicate that LVI functions serve as a separate indicator of life for patients with breast tumors who have received neoadjuvant chemotherapy. Furthermore, researchers have found a strong association between the basal-like subtype and the luminal B subtype with HER2(-) and LVI [20]. Cheung and Choi et al. concluded that diagnostic use of Initial DWI (diffusion-weighted imaging) and MR imaging (MRI) assists in the detection of LVI in individuals diagnosed with breast cancer [21,22].

Multivariate analysis of the current study revealed that cases that exhibit LVI have the prevalence of remaining positive axillary lymphatic nodes had the highest direct relation (odds ratio [OR], 6.438; p < 0.05) among the subgroup of individuals with hormone receptor-positive (HR+) status. We suggest that future researchers focus on further enhancing the efficacy of LVI as a prognostic indicator and predictor of recurrence. In conclusion, examining tumor biology has become a crucial element in predicting the pathological response for the axillary and breast regions. The current investigation revealed that attaining axillary pathological complete response (pCR) posed a formidable challenge for patients characterized by HER2-negative status, Tumours of histological type III, and low levels of Ki-67 expression. This outcome lines up with prior research findings [15]. The current study depicts no discernible effect on axillary pathological complete response (pCR) regardless of particular chemotherapy regimens. In brief, our prediction model, which incorporates imaging and pathology data, exhibited a satisfactory capacity to differentiate between axillary pathological complete response (pCR) and the existence of residual positive axillary lymph nodes. Simultaneously, the test group exhibits a value of 0.847, while the validity group demonstrates a value of 0.813, indicating the model's competence and effectiveness.

In the present study, researchers conducted a multivariate analysis to evaluate the risk of the parameters above for axillary pathological complete responses. Individuals exhibiting risk scores ranging from 0 to 2 are categorized as low-risk, while individuals having scores of 3 to 6 indicated high risk. The group classified as low-risk reported a pathological complete response (pCR) rate of 29.6% (42 out of 142), whereas the group classified as high-risk exhibited a pCR rate of 4.0% (4 out of 101). Our findings indicate that the assigned scores of our model exhibit excellent performance for both low and high-risk categories. The Kaplan-Meier curve assessment in the context of survival revealed that lymphatic node condition following NAC did not display a statistically significant correlation with overall survival (OS). Nevertheless, previous research has demonstrated a substantial association between lymph node status and disease-free survival (DFS) (P = 0.048). The observed association between survival and diseased stage (P < 0.05) implies the presence of potential factors, such as limited sample size and variability in postoperative care, that may impact this correlation.

The current methodologies for evaluating axillary lymph nodes encompass physical examination and ultrasound imaging techniques. Researchers have posited that lymph node staging before NAC enables medical professionals to make informed judgments regarding local treatment, minimizing the potential for undertreatment [23]. Nevertheless, the cohort of individuals exhibiting positive lymph nodes in a clinical setting and undergoing axillary biopsy was tiny. Research findings imply that the sensitivity of the biopsy needle biopsy was only 25% [24], and around 50% of axillary infiltration in women was successfully detected before surgery [25]. Axillary lymph node dissection was still needed, though certain patients did attain axillary pathological complete response following neoadjuvant chemotherapy. The study's findings indicated no statistically significant difference in the survival rate between individuals with positive axillary lymph nodes and those without (P > 0.05). Performing axillary lymph node dissection (ALND) in cancer patients without medical indication might lead to an increased incidence of axillary damage, ultimately giving rise to post-surgical complications such as lymphedema and diminished muscular strength. Therefore, our model has the potential to guide the management and treatment of the axilla, thereby minimizing the need for unneeded radiation therapy and associated consequences.

The present study has specific limitations. Despite the presence of ultrasonic intervention experts with top professional credentials, the occurrence of false negatives remains inevitable. Secondly, our methodology is specifically applicable for assessing axillary pathological complete response (pCR) rather than breast pCR. Thirdly, the assessment of HER2 expression using preoperative puncture may not correspond with the results obtained from postoperative pathology, mostly due to the presence of inherent tumor heterogeneity. The study's sample size was limited, and the study design was retrospective rather than prospective. It is essential to consider these factors when incorporating the study into future prospective investigations.

## Conclusions

In conclusion, results revealed that the rate of mastectomy decreased after introducing the neoadjuvant therapy. Furthermore, results indicated that individuals who had undergone neoadjuvant chemotherapy and still had positive axillary lymph nodes reported a higher likelihood of exhibiting suspected nodules during ultrasound examination before surgery. Our research introduces a novel predictive model that combines imaging and pathology data to help patients and healthcare professionals assess the effectiveness of NAC for hormone-receptor-positive breast tumors. The model holds particular significance for individuals who exhibit clinical positivity in their LNs. Consequently, the model has the potential to provide guidance for the management of axillary lymph nodes and prevent unnecessary dissection.
